# Supplementary material for: Ballistic supercavitating nanoparticles driven by single Gaussian beam optical pushing and pulling forces
Source: Nat Commun. 2020 May 15;11:2404. doi: 10.1038/s41467-020-16267-9 (PMC7228977; doi:10.1038/s41467-020-16267-9)
Supplement: Supplementary file 1 — Description of Additional Supplementary Files [file 41467_2020_16267_MOESM1_ESM.docx]

**Description of Additional Supplementary Files**

**File name:** Supplementary Video 1

**Description:** Ultra-fast ballistic Au-silica core-shell NPs. Power of laser = 690 mW; 20× objective lens for the laser; NP number density: 4×1014 # m-3; field of view size: 1684 µm × 194 µm. The Gaussian beam propagates from left to right. Time is in second.

**File name:** Supplementary Video 2

**Description:** Ultra-fast ballistic Au-silica core-shell NPs with positive motion. Power of laser = 690 mW; 20× objective lens for the laser; NP number density: 4×1014 # m-3 ; field of view size: 657 µm × 194 µm. The Gaussian beam propagates from left to right. Frame number is shown.

**File name:** Supplementary Video 3

**Description:** Ultra-fast ballistic Au-silica core-shell NPs with negative motion. Power of laser = 690 mW; 20× objective lens for the laser; NP number density: 4×1014 # m-3; field of view size: 657 µm × 194 µm. The Gaussian beam propagates from left to right. Frame number is shown.

**File name:** Supplementary Video 4

**Description:** Ultra-fast ballistic Au-silica core-shell NPs with negative motion in a low NP concentration suspension. Power of laser = 690 mW; 10× objective lens for the laser; NP number density: 5×1013 # m-3 ; field of view size: 254 µm × 98 µm. The Gaussian beam propagates from left to right. Time is in second.

**File name:** Supplementary Video 5

**Description:** Ultra-fast ballistic Au-silica core-shell NPs with positive motion in a low NP concentration suspension. Power of laser = 690 mW; 20× objective lens for the laser; NP number density: 1×1013 # m-3; field of view size: 505 µm × 57 µm. The Gaussian beam propagates from left to right. Time is in second.

**File name:** Supplementary Video 6

**Description:** Ultra-fast ballistic Au nanorod NPs. Power of laser = 1 W; 20× objective lens for the laser; NP number density: 1.3×1017 # m-3 ; field of view size: 1114 µm × 104 µm. The Gaussian beam propagates from left to right. Time is in second.
