# Supplementary material for: Ballistic supercavitating nanoparticles driven by single Gaussian beam optical pushing and pulling forces
Source: Nat Commun. 2020 May 15;11:2404. doi: 10.1038/s41467-020-16267-9 (PMC7228977; doi:10.1038/s41467-020-16267-9)
Supplement: Supplementary file 8 — Supplementary Information [file 41467_2020_16267_MOESM8_ESM.pdf]

Supplementary Information for

Ballistic Supercavitating Nanoparticles Driven by Single Gaussian Beam Optical Pushing and Pulling forces

Supplementary Note 1: Optical Force Field on a SiO_2-Au Core-Shell NP under the Gaussian Beam

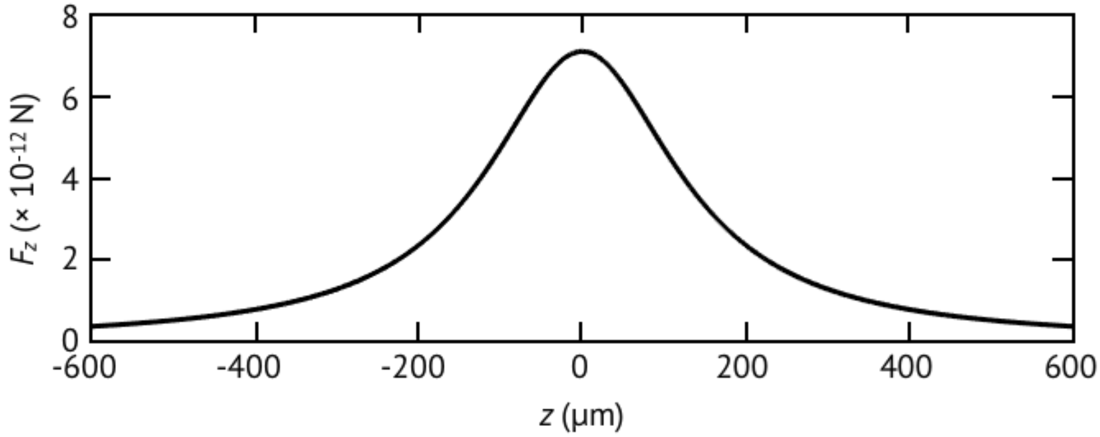

Supplementary Figure 1| The calculated $\mathbf{F_z}$ on a SiO_2-Au core-shell NP along the central axis (z-direction) of the Gaussian beam with the beam waist of 6 μm and the intensity of 12 mW μm^{-2} at the focal plane (z = 0).

Supplementary Note 2: Deposited Ballistic NPs on Quartz Substrates

We have leveraged the uniqueness of the negative motion of NPs to avoid the deposition of non-supercavitating NPs, as the optical pulling force on an NP is only made possible with supercavitation. As the SEM image in Supplementary Figure 2b shows, the NPs on the surface of the quartz are single NPs and their sizes are well-defined.

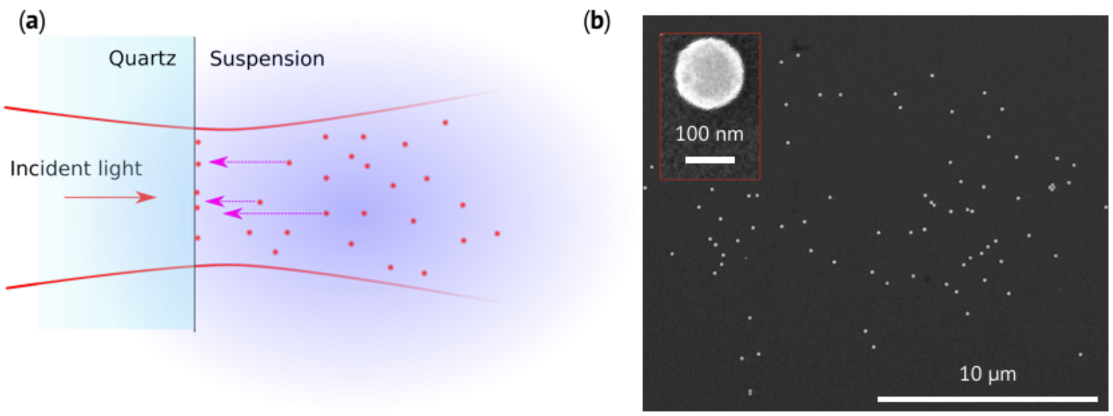

Supplementary Figure 2| (a) An experimental setup to deposit the ballistic NPs with negative motion on a quartz substrate. An objective lens (10×, beam waist ~20 μm) was used to focus the femtosecond laser with a power of 690 mW on the substrate surface. (b) The collected ballistic NPs on the quartz substrate visualized using SEM. The inset is the magnified SEM image of an Au NP on the quartz.

Supplementary Note 3: Temperature of Plasmonic Au NP in a Nanobubble

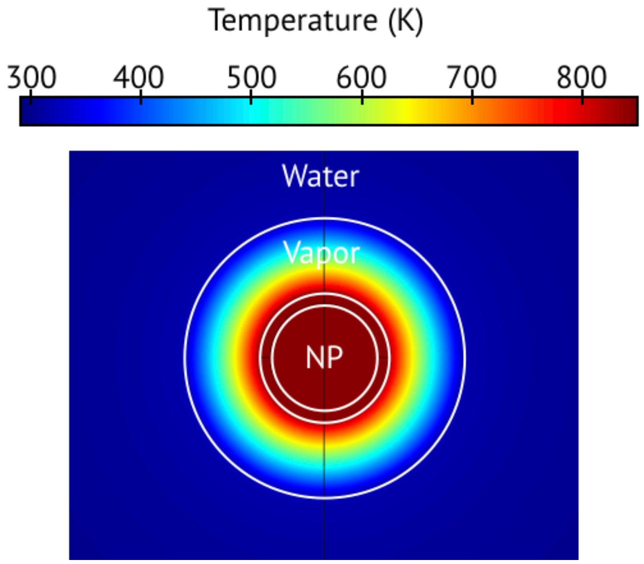

Supplementary Figure 3| The calculated temperature of the NP-in-nanobubble system when the amplitude of the electric field of an incident planewave is 2.6×10^{6} V m^{-1}, corresponding to the experimental laser condition.

Supplementary Note 4: Pump-Probe Optical Scattering Imaging Experiment and Formation of Nanobubble

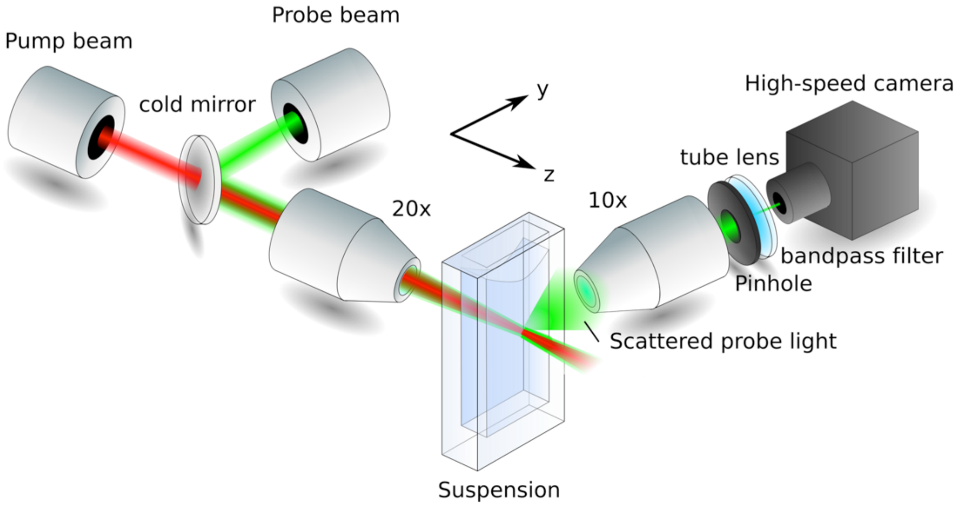

Supplementary Figure 4| Schematic of experimental setup to detect nanobubble formation on Au NPs.

Supplementary Figure 4 shows the schematic of the experimental setup of a pump-probe optical scattering imaging method. In this method, we use a pump light (femtosecond pulsed laser, wavelength of 800 nm, repetition rate of 80.7 Mhz, and power of 690 mW) and a probe light (continuous laser, wavelength of 533 nm, and power of ~ 5 mW). The pump light wavelength overlaps with the surface plasmon resonance (SPR) of Au NPs in water, where an Au NP consists of a silica core (~100 nm) and an Au shell (~10 nm), which is the same Au NP used in the NP movement experiment. The wavelength of the probe light is off the plasmonic resonance of the Au NPs in water. The pump light and the probe light pass through an objective lens (20×) and then are introduced as Gaussian beams in the suspension of Au NPs in water as shown in Supplementary Figure 4, where the suspension is contained in a quartz cuvette with 4 windows (10 mm × 10 mm). The optical axes of Gaussian beams are parallel to the z-axis. On the other hand, there is another objective lens (10 ×) for detection, which is aligned with the *y*-axis. The 10× objective lens focuses on a cross-plane in the suspension where the optical axes of the pump beam and the probe beam overlaps. The scattered lights from the cross-plane sequentially pass through the 10× objective lens, a mechanical pinhole, a low-frequency bandpass filter, a tube lens, and then is focused onto the image sensor of a high-speed camera with ultra-high light sensitivity

(HX-7, color ISO rating of 8000). The mechanical pinhole works as an optical aperture so as to allow the scattered lights at a certain solid angle to pass through, enhancing the sensitivity[1]. The low-frequency bandpass filter blocks any scattered pump light and only allows scattered probe lights to be pass through.

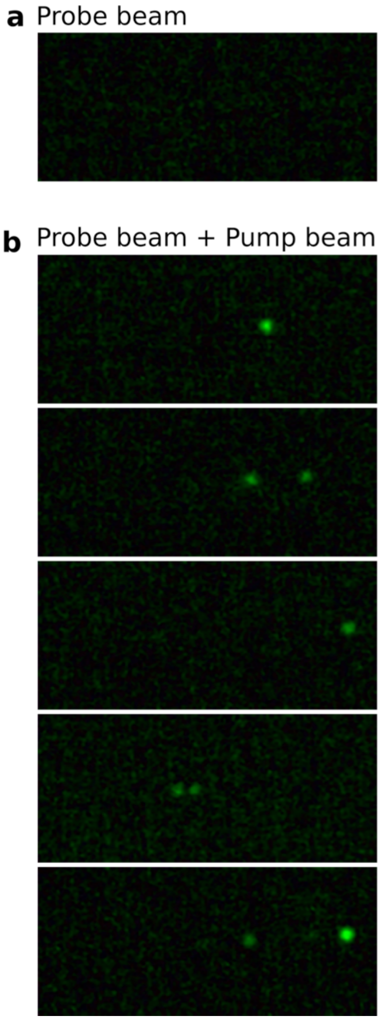

Supplementary Figure 5| Images from the optical scattering imaging experiment for (**a**) without pump beam and (**b**) with pump beam; each green spot corresponds to the diffraction limited scattered probe light from the location of the Au NP with nanobubble.

The optical scattering imaging method in Supplementary Figure 4 is capable of detecting the intensity change of the scattered probe light at a certain solid angle on the cross-plane when the pump beam is illuminated at Au NPs in the suspension. We first find that without the pump beam

the camera cannot detect any noticeable scattered probe light (Supplementary Figure 5a). With the pump beam, it is clearly seen that diffraction limited green spots appear on the cross-plane (Supplementary Figure 5b). This shows the far-field spatial intensity profile of scattered probe light from Au NPs is strongly changed under the pump beam. Such modification of the scattered light is only possible when the Au NP is near-field coupled to object(s) that is(are) located on (or at close vicinity of) the Au NP within subwavelength length scale. The most reasonable interpretation for the object(s) is the formation of nanobubbles on Au NPs, which agrees well with previous results demonstrating the formation of nanobubbles on plasmonic NPs using similar optical scattering imaging technique[2,3].

Supplementary Note 5: Approximation of the Time-averaged Optical Force from the Pulsed Laser with the Force from a Continuous Single Plane Wave

The optical force by a pulsed laser can be a function of time, and it can be described by Lorentz's force density ($\mathbf{f}$) equation on an object:

$$\mathbf{f} - \frac{\partial \mathbf{S}}{\partial t} = \nabla \cdot T \qquad \text{Equation 1}$$

where T is the Maxwell stress tensor, and $\mathbf{S}$ is the electromagnetic momentum density (e.g., $\mathbf{D} \times \mathbf{B}$ by Minkowski momentum or $(\mathbf{E} \times \mathbf{H})/c^2$ by Abraham momentum)[4–6]. It is clear that $\mathbf{f}$ can be a function of time (t) if the incident light is a function of time. When the incident light is a pulsed beam, the amplitude oscillation of the electromagnetic field at the optical frequency is convoluted by the duration of a pulse. In our case, the duration is ~ 94 fs, and the optical frequency is 3.7×10^{14} Hz (= a period of ~2.7 fs). These time scales are much faster than the mechanical response of the ballistic NPs. For example, at ~ 94 fs, a ballistic NP with a velocity of 100,000 μm s^{-1} can only move ~$O(10^{-14})$ m. This suggests that averaging the force in time should be appropriate for analyzing the motion of NPs. These are well described in elsewhere[7–9]. Using Supplementary Equation 1, the time-averaged force $\langle \mathbf{F} \rangle_t$ on an object can be written as:

$$\langle \mathbf{F} \rangle_t = \frac{1}{t_2 - t_1} \left\{ \int_{t_1}^{t_2} \oint_A T \cdot \mathrm{d}A \, \mathrm{d}t - \int_V \mathbf{S}(t = t_2) \, \mathrm{d}V + \int_V \mathbf{S}(t = t_1) \, \mathrm{d}V \right\} \qquad \text{Equation 2}$$

where A and V are the surface and the volume of the object, respectively. Since the pulsed laser has a repetition rate of ν_0 = 80.7 MHz, if we pick t_1 and t_2 to integrate over one pulse, the last two volume integral terms in Supplementary Equation 2 vanish as they are identical. It means that the momentum density terms are not needed from the perspective of the time-averaged force, and the Maxwell stress tensor alone is sufficient to determine $\langle \mathbf{F} \rangle_t$. Then, we check if $\langle \mathbf{F} \rangle_t$ from a pulsed laser is the same as that from a continuous laser with the same power density. To check this, we define the pulsed incident light as the summation of dispersive planewaves, where each planewave is linearly polarized in the x-direction and propagates in the z-direction:

$$\mathbf{E}(t, z) = \mathbf{x} \frac{1}{\nu_0} \int_{-\infty}^{+\infty} E_\nu(\nu) \mathrm{e}^{i(2\pi\nu)(t - \frac{n_\mathrm{m} z}{c_0})} \, \mathrm{d}\nu \qquad \text{Equation 3}$$

where $\mathbf{E}(z, t)$ is the electric field as a function of space and time, c_0 is the speed of light in vacuum, and n_m is the refractive index of the medium. $\mathbf{E}_\nu(\nu)$ is the amplitude of electric field as a function of frequency (ν) and can be expressed as a Gaussian function:

$$E_\nu(\nu) = E_0 \sqrt{\nu_0} \left(\frac{2\pi}{a} \right)^{\frac{1}{4}} \mathrm{e}^{-\left(\frac{\pi^2 (\nu - \frac{\omega_\mathrm{c}}{2\pi})^2}{a} \right)} \qquad \text{Equation 4}$$

where ω_c is the central mode angular frequency of the pulsed light (2.35×10^{15} Hz, corresponding to a vacuum wavelength of 800 nm), $a = 2\ln 2 \, \tau^{-2}$, and E_0 is a coefficient in the unit of V m^{-1}, which gives the time-averaged power flux of a pulse as $\frac{1}{2} c_0 \epsilon_0 n_\mathrm{m} E_0^2$. Since the duration of the laser in our experiment is ~ 94 fs, which is an ultra-short pulse, the time-averaged force $\langle \mathbf{F} \rangle_t$ can be approximated as the summation of the forces from every planewave components in the pulsed light. Subsequently, Supplementary Equation 2 can be re-written with Supplementary Equation 4 as:

$$\langle \mathbf{F} \rangle_t = \frac{1}{t_2 - t_1} \int_{t_1}^{t_2} \oint_A T \cdot \mathrm{d}A\, \mathrm{d}t \approx \int_{-\infty}^{+\infty} \frac{|E_\nu(\nu)|^2}{\nu_0} \mathbf{F}(\nu) \mathrm{d}\nu \qquad \text{Equation 5}$$

where $\mathbf{F}(\nu)$ is the optical force when a single planewave at the frequency of ν with the unity amplitude of electric field is incident to the object, and it can be calculated by $\oint_A \langle T \rangle_t \cdot \mathrm{d}A$; $\langle T \rangle_t$ is the time-averaged Maxwell stress tensor given by the single planewave; $\mathbf{F}(\nu)$ is in the unit of N per V m^{-2}. Thus, we can estimate $\langle \mathbf{F} \rangle_t$ by calculating $\mathbf{F}(\nu)$ in the frequency domain. Supplementary Figure 6 shows $|E_\nu(\nu)|^2(\nu_0)^{-1}$ and $\mathbf{F}(\nu)$ as a function of ν, where E_0 is 2.6×10^6 V m^{-1} corresponding to the light intensity in our experiment. To study the positive and negative motions of ballistic NPs, representative cases are chosen to calculate $\mathbf{F}(\nu)$. These include r_{nb} = 130 nm and θ = 180° and r_{nb} = 130 nm and θ = 0° (refer to Figure 2 in main text for coordinates). Supplementary Equation 5 gives $\langle \mathbf{F} \rangle_t$ as 1.413 × 10^{12} N for the case of r_{nb} = 130 nm and θ = 180° and -7.553 × 10^{13} N for the case of r_{nb} = 130 nm and θ = 0°. These are almost identical to the optical forces given by the continuous planewave at a central frequency of $\frac{\omega_c}{2\pi}$ = 3.7474 × 10^{14} Hz (or λ = 800 nm), which are 1.411 × 10^{12} N for the case of r_{nb} = 130 nm and θ = 180° and -7.554 × 10^{13} N for the case of r_{nb} = 130 nm and θ = 0°. These results ensure that the time-averaged optical force by the pulsed light can be approximated as the optical force from a continuous wave light with the same central frequency and power density.

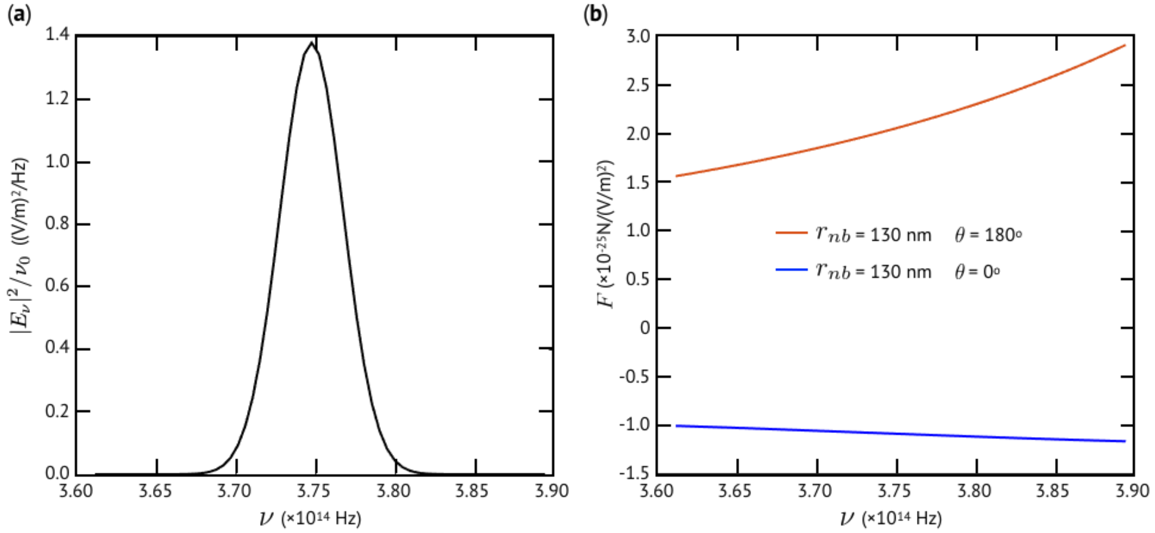

Supplementary Figure 6| The calculated (a) $|E_{\nu}(\nu)|^2\,(\nu_0)^{-1}$ and (b) $\mathbf{F}(\nu)$ as a function of ν when E_0 is 2.6×10^6 V m^{-1}, corresponding to our laser in the experiment.

Supplementary Note 6: Optical Stress Tensor Profile on the Surface of NP in Nanobubble

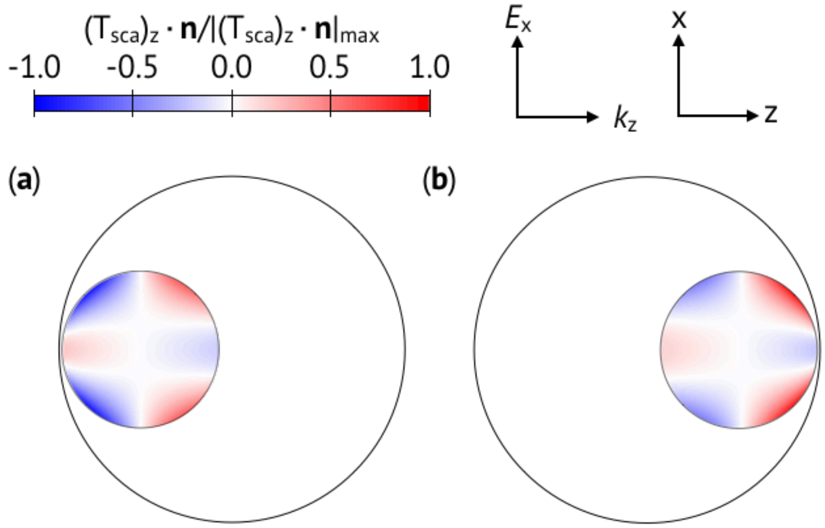

Supplementary Figure 7. The normalized stress tensor profile from the scattered fields of cases with (a) r_{nb}=130 nm and θ=0° and (b) r_{nb}=130 nm and θ=180°. Here, **n** depicts a normal vector at the surface of Au NP.

Supplementary Note 7: Far-Field Scattering Pattern of NP in Nanobubble

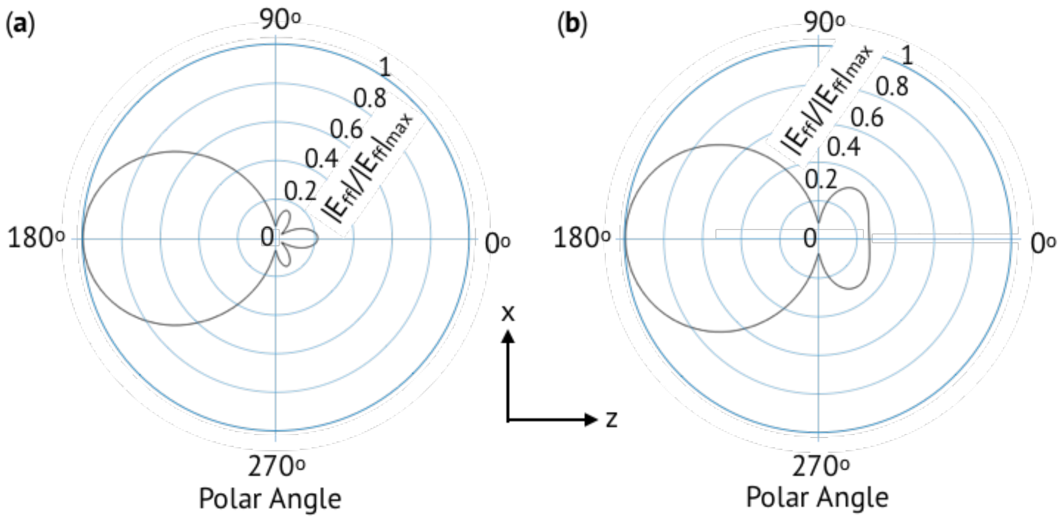

Supplementary Figure 8. The far-field scattering patterns of the cases with (a) r_{nb}=130 nm and θ=0° and (b) r_{nb}=130 nm and θ=180°. Here, the normalized scattering electric field amplitude ($\frac{|\mathbf{E}_{\mathrm{ff}}|}{|\mathbf{E}_{\mathrm{ff}}|_{\max}}$) in the far-field domain is plotted as a function of the polar angle in the *x*-*z* plane at y = 0. $|\mathbf{E}_{\mathrm{ff}}|_{\max}$ is the maximum of $|\mathbf{E}_{\mathrm{ff}}|$ at the polar angle of 180°. The location of NP being encapsulated by the nanobubble is at the center of the polar coordinate. Each circle in the polar coordinate corresponds to an isovalue of $\frac{|\mathbf{E}_{\mathrm{ff}}|}{|\mathbf{E}_{\mathrm{ff}}|_{\max}}$.

Supplementary Note 8: Continuous Evaporation of Nanobubble Front by Hot and Moving Nanoparticle using Molecular Dynamics Simulations

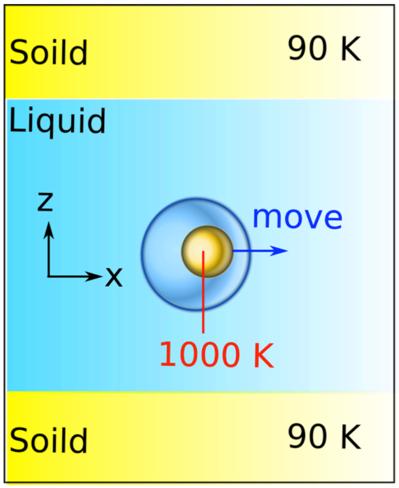

Supplementary Figure 9| Schematic a moving NP encapsulated by a nanobubble in liquid used for the MD simulations.

In this part, we use molecular dynamics (MD) simulations to verify the hypothesis that the laser excited hot NP can extend the boundary of a nanobubble encapsulating it. In the main text, we assume that: as the super-cavitating Au NP moves forward, it can continuously evaporate water molecules, keeping a vapor environment in front of it, and expanding the vapor/liquid boundary forward. The simulation domain used for MD simulations consists of a nanoparticle (288 solid atoms with the potential mimicking platinum) immersed in liquid (173276 liquid argon atoms),

which is confined between two solid walls (72828 solid platinum atoms) as shown in Supplementary Figure 9. The simulation box has a dimension of 20 nm (in x) × 20 nm (in y) × 24 nm (in z), and has periodic boundary conditions in all three directions. The Lennard-Jones (L-J) potential4 is employed to simulate the interactions between the particles:

$$E_{ij}^{\mathrm{LJ}} = 4\varepsilon_{ij}\left[(\frac{\sigma_{ij}}{r_{ij}})^{12} - (\frac{\sigma_{ij}}{r_{ij}})^{6}\right] \quad \text{Equation 6}$$

where ε_{ij} and σ_{ij} are the respectively the energy and length constants, r_{ij} is the distance between two atoms, i and j. The L-J potential parameters for argon-argon and argon-platinum used in this study are: $\varepsilon_{Ar-Ar} = 0.0104$ eV, $\sigma_{Ar-Ar} = 3.40$ Å, $\varepsilon_{Pt-Pt} = 0.52$ eV, $\sigma_{Pt-Pt} = 2.475$ Å, $\varepsilon_{Pt-Ar} = 0.02$ eV, $\sigma_{Pt-Ar} = 2.94$ Å. A cutoff of 13.5 Å is used for the L-J interactions. The timestep is chosen as 5 fs which can reliably simulate the dynamics of the system without energy drifting[10]. The visualization is done by OVITO (Open Visualization Tool)[11].

The system is first equilibrated in a canonical ensemble (NVT) at 90 K for 2.5 ns. After the structures are fully relaxed, the microcanonical ensemble (NVE) is applied to the liquid particles for 5 ns. The temperature of the solid walls is kept at 90 K (much less than the critical temperature of the fluid), which prevents the overall heating of the whole system. This also effectively mimics an isolated NP condition in the dilute limit since the inter-particle distance is around 10-40 μm in our experiments. The temperature of the solid NP is thermostated at 1000 K (higher than the critical temperature of the fluid). We set this temperature because it was reported that Au NP in encapsulated by a vapor bubble can have temperature over 1000 °C under pulsed laser irradiation[12]. The volume of liquid is divided into $50 \times 50 \times 50$ voxels. In each voxel, the number of atoms is spatially averaged, which is then used to calculate the number density for that voxel[13]. A nanobubble is considered to form if the number density is less than the critical density of argon liquid. Here, the critical density of liquid argon is 0.536 g cm^{-3}, which is determined from Ref.[14]. After the creation of a nanobubble around the hot NP, we start to move the NP. Semi-isotropic Berendsen pressure coupling is used to keep the z-direction pressure constant at 2 bars, which is higher than the triple point pressure and at the same time lower than the critical pressure. As shown in Figure 3c in the main text, it is clearly seen that the NP continuously evaporates the liquid

molecules in front of it when it moves forward, so that the nanobubble boundary extends in the moving direction of NP. This speed, as well as the corresponding body-length speed, is larger than the observed Au NP moving speeds. With smaller speed, the bubble extension around the moving NP will be even easier. This MD result supports the Leidenfrost effect hypothesis of ballistic Au NP at the atomistic level.

Supplementary Note 9: Constant Temperature Assumption of Au NP in MD Simulation

We assumed the temperature of the NP as a constant in the MD simulation in Supplementary Note 8. Here, we give the rationale behind this assumption. In the experiment, we use the femtosecond pulsed laser, and accordingly the volumetric heat generation in the Au NP by the SPR should be periodic. This possibly makes the temperature of Au NP in a nanobubble decrease in the window of time interval between two laser pulses. Therefore, it is important to check how much the temperature of an Au NP decreases during this time interval (here it is 12.3 ns corresponding to our laser repetition rate of 80.7 MHz). Since we are interested in the hot Au NP encapsulated by a nanobubble, it is reasonable to assume the temperature of the Au NP at t = 0 is at 1000 K according to Ref.[12]. For the simplicity, we consider two-extreme case: one is the Au NP in gas and the other is in liquid water. The temperature drop of Au NP in a nanobubble in water at t = 12.3 ns should be between these two-extreme cases. In this case, the temperature (T) of the Au NP can be given by Newton's law of cooling as:

$$T = T_{\infty} + (T_{t=0} - T_{\infty})\exp\left(-\frac{hA}{C_{\mathrm{np}}}t\right) \qquad \text{Equation 7}$$

where T_{∞} is the temperature in the bulk of water at the room temperature (300 K), C_{np} is the heat capacity of Au NP, A is the surface area of Au NP, and h is the heat transfer coefficient of gas or liquid water: 32 J s^{-1} m^{-2} K^{-1} for gas (air) and 6000 J s^{-1} m^{-2} $K^{-1}$for water. Here, the Au NP consists of a silica core with the radius of 50 nm and an Au shell with the thickness of 10 nm. We assume C_{np} is the summation of the heat capacitance of Au shell (C_{Aushell}) and that of silica core (C_{sio2core}): C_{Aushell} = 9.24×10^{-16} J K^{-1} and $C_{\mathrm{SiO_2core}}$ = 8.56×10^{-16} J K^{-1}. As a result, at t = 12.3 ns, Supplementary Equation 7 gives us T = 999.9930 K for the case of Au NP in gas and T = 998.6885

K for the case of Au NP in liquid water. In another word, the temperature of the NP barely changes between two laser pulses. These estimations support the assumption that the temperature of NP in MD simulations is constant.

Supplementary Note 10: Super-fast Ballistic Movement of Au Nanorods

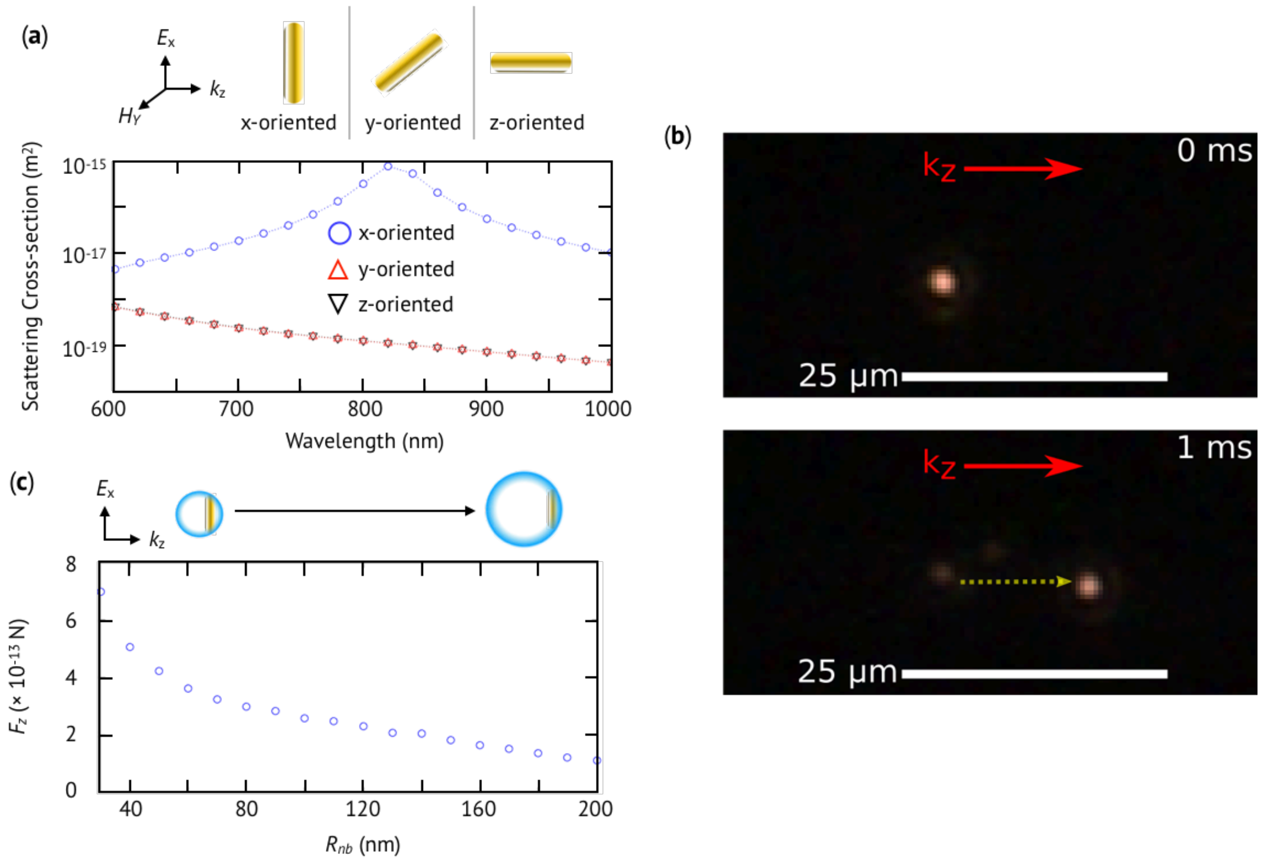

Supplementary Figure 10| (**a**) The calculated scattering cross-section of an Au nanorod (NR) when it is aligned with the *x*, *y*, or *z*-axis. Only when the Au NR is aligned with the x-axis the SPR is possible. (**b**) Dark-field optical images of a ballistic Au NR with a positive motion. (**c**) The calculated optical force in the *z*-direction ($\mathbf{F}_z$) on the *x*-oriented Au NR as a function of the radius of nano-bubble (R_{nb}). Here, the amplitude of $\mathbf{E}_x$ is 2.6×10^6 V m^{-1}. The insets illustrate the schematic configurations of Au NR with a nano-bubble with different R_{nb}.

We perform an experiment with Au nanorod (NR) to check whether the observed ballistic movement is potentially generalizable. The Au NR has a length of 48 nm and a width of 12 nm. The Au NR in water can have an SPR peak at the wavelength (λ) of 800 nm when the axis of NR is parallel to the polarization direction of the incident light, which is clearly shown in Supplementary Figure 10a. It indicates that the Au NR can be intensely excited only when aligning with the field direction of the 800-nm-wavelength laser. The Au NRs are dispersed in water with the number density of 1.3×10^{17} # m^{-3}. We use the same experimental setup described in Figure 1a in the main text to observe NR motion. A femtosecond pulsed laser with a power of ~1 W passes through a 20× objective lens, introducing a Gaussian beam in the Au NR-water suspension. Since the SPR characteristic of Au NR is anisotropic due to the shape of the rod, only the Au NR whose longitudinal axis is well aligned with the direction of incident electric field can have the SPR and be heated to create supercavitation. This implies that there is a low probability to observe ballistic Au NRs, because slight Au NR misalignment with the electric fields may interrupt the heating and thus supercavitation. Although rare, in Supplementary Figure 10, we could still observe a glowing dot moving over a distance of ~13 μm within 1 ms almost perfectly along the beam propagating direction (see also Supplementary Video 6). This ballistic Au NR shows the normalized speed of 260,000 body-length s^{-1}, which is ~10^2-10^5 times faster than the reported nano/micro swimmers driven by optical forces[15,16]. The magnitude of the optical force has the range of 1×10^{-13} N ~ 7×10^{-13} N and monotonically decreases as the size of nanobubble increases, as calculated using EM wave simulation (Supplementary Figure 10c). Balancing the averaged magnitude of optical forces with the Stokes' drag force can yield a dynamic viscosity of 4.4×10^{-5} kg $m^{-1}s^{-1}$, which is much lower than that of liquid water and on the same order of magnitude as that of vapor. This result demonstrates that the observed ballistic NP movement can be potentially generalized to other kinds of plasmonic NPs with different geometry, composition and dimensions as long as the SPR heating can be excited.

Supplementary References

1. Lachaine, R., Boulais, E., Bourbeau, E. & Meunier, M. Effect of pulse duration on plasmonic enhanced ultrafast laser-induced bubble generation in water. *Appl. Phys. A* **112**, 119 - 122 (2013).
2. Lukianova-Hleb, E. *et al.* Plasmonic Nanobubbles as Transient Vapor Nanobubbles Generated around Plasmonic Nanoparticles. *ACS Nano* **4**, 2109 - 2123 (2010).
3. Lapotko, D. Optical excitation and detection of vapor bubbles around plasmonic

nanoparticles. *Opt. Express* **17**, 2538 - 2556 (2009).
4. Hinds, E. A. & Barnett, S. M. Momentum exchange between light and a single atom: Abraham or Minkowski? *Phys. Rev. Lett.* **102**, 050403 (2009).
5. Scalora, M. *et al.* Radiation pressure of light pulses and conservation of linear momentum in dispersive media. *Phys. Rev. E - Stat. Nonlinear, Soft Matter Phys.* **73**, 056604 (2006).
6. Mansuripur, M. & Zakharian, A. R. Maxwell's macroscopic equations, the energy-momentum postulates, and the Lorentz law of force. *Phys. Rev. E - Stat. Nonlinear, Soft Matter Phys.* **79**, 026608 (2009).
7. Ellingsen, S. Å. Theory of microdroplet and microbubble deformation by Gaussian laser beam. *J. Opt. Soc. Am. B* **30**, 1694 - 1710 (2013).
8. Nieminen, T. A. *et al.* Optical tweezers: Theory and modelling. *J. Quant. Spectrosc. Radiat. Transf.* **146**, 59–80 (2014).
9. Preez-Wilkinson, N. du, Stilgoe, A. B., Alzaidi, T., Rubinsztein-Dunlop, H. & Nieminen, T. A. Forces due to pulsed beams in optical tweezers: linear effects. *Opt. Express* **23**, 7190 - 7208 (2015).
10. *Understanding Molecular Simulation*. (Elsevier, 2002). doi:10.1016/B978-0-12-267351-1.X5000-7
11. Stukowski, A. Visualization and analysis of atomistic simulation data with OVITO-the Open Visualization Tool. *Model. Simul. Mater. Sci. Eng.* **18**, 015 012 (2010).
12. Hodak, J. H., Henglein, A., Giersig, M. & Hartland, G. V. Laser-Induced Inter-Diffusion in AuAg Core-Shell Nanoparticles. *J. Phys. Chem. B* **104**, 11708–11718 (2000).
13. Maheshwari, S., van der Hoef, M., Prosperetti, A. & Lohse, D. Dynamics of Formation of a Vapor Nanobubble Around a Heated Nanoparticle. *J. Phys. Chem. C* **122**, 20571–20580 (2018).
14. Bale, H. D., Dobbs, B. C., Lin, J. S. & Schmidt, P. W. X-ray scattering studies of critical opalescence in argon at constant density. *Phys. Rev. Lett.* **25**, 1556–1559 (1970).
15. Königer, A. & Köhler, W. Optical Funneling and Trapping of Gold Colloids in Convergent Laser Beams. *ACS Nano* **6**, 4400–4409 (2012).
16. Kajorndejnukul, V., Ding, W., Sukhov, S., Qiu, C.-W. & Dogariu, A. Linear momentum increase and negative optical forces at dielectric interface. *Nat. Photonics* **7**, 787–790 (2013).
